# Rapid onset of functional tic-like behaviors among adolescent girls—Minnesota, September–November 2021

**DOI:** 10.3389/fneur.2022.1063261

**Published:** 2023-01-19

**Authors:** Melanie J. Firestone, Stacy Holzbauer, Christine Conelea, Richard Danila, Kirk Smith, Rebecca H. Bitsko, Susan M. Klammer, Stefan Gingerich, Ruth Lynfield

**Affiliations:** ^1^Epidemic Intelligence Service, Centers for Disease Control and Prevention (CDC), Atlanta, GA, United States; ^2^Minnesota Department of Health, St. Paul, MN, United States; ^3^Division of State and Local Readiness, Center for Preparedness and Response, Centers for Disease Control and Prevention (CDC), Atlanta, GA, United States; ^4^Department of Psychiatry and Behavioral Sciences, University of Minnesota Medical School, Minneapolis, MN, United States; ^5^Division of Human Development and Disability, National Center on Birth Defects and Developmental Disabilities, Centers for Disease Control and Prevention (CDC), Atlanta, GA, United States

**Keywords:** tics and Tourette Syndrome, mental health, adolescent girls, functional neurologic disorders, tic disorder

## Abstract

**Background:**

On October 15, 2021, the Minnesota Department of Health began investigating a school cluster of students experiencing tic-like behaviors thought to be related to recent COVID-19. The objective of this report is to describe the investigation, key findings, and public health recommendations.

**Methods:**

Affected students and proxies were interviewed with a standardized questionnaire including validated depression and anxiety screens.

**Results:**

Eight students had tic-like behaviors lasting >24 h after initial report with onset during September 26–October 30, 2021. All eight students were females aged 15–17 years. All students either had a history of depression or anxiety or scored as having more than minimal anxiety or depression on validated screens. Four students previously had confirmed COVID-19: the interval between prior COVID-19 and tic symptom onset varied from more than a year prior to tic symptom onset to at the time of tic symptom onset.

**Conclusion:**

The onset of tic-like behaviors at one school in Minnesota appeared to be related more to underlying mental health conditions than recent COVID-19. These findings highlight the need to better understand functional tic-like behaviors and adolescent mental health.

## Key terms

- Functional tic-like behaviors.
- Tic disorders.
- Mental health.
- Functional neurological disorder.

## Introduction

Since the COVID-19 pandemic began, clinicians have reported an increase in the number of young persons seeking medical care for tics (1–8). This increase encompasses several patient profiles: (1) patients with known history of tic disorder (TD), such as Tourette Syndrome (TS) who are experiencing exacerbations of existing tics, often alongside increased psychosocial stress, a known contributor to tic exacerbation and diminished tic controllability (9, 10) (2) patients with known or strongly suspected TD history experiencing an increase or rapid onset of complex tics; and (3) patients, typically adolescents, with no recognized TD history experiencing rapid onset of often complex, tic-like behaviors for the first time (5). Although sudden development of tic-like behaviors have been seen before the COVID-19 pandemic (11), it is hypothesized that increased exposure to tic-like behaviors *via* social media (3, 4) and other stressors have contributed to increases in tic-like behaviors.

Clinical differentiation of these patient profiles can be challenging. Symptoms experienced across these groups share many phenomenological similarities (e.g., topography, frequency, intensity of tics/tic-like behaviors, susceptibility of these behaviors to mimicking or “echo phenomena”), and a positive history of tics can be difficult to confirm retrospectively. However, some distinct characteristics have been observed, suggesting divergent underlying mechanisms in the third group of patients with new rapid-onset tic-like behaviors, relative to TD or TS patients.

TDs are neurodevelopmental disorders that typically begin in early childhood and are characterized by motor or vocal tics (5). The median age of diagnosis for TDs is 7 years (12). Peak severity usually occurs during the ages of 10 and 12 years (13), followed by gradual attenuation of tics in approximately two-thirds of patients, likely due to functional and structural maturation of the prefrontal-striatal network enabling improved top-down control of tics (14). TS occurs more often in boys than girls at a ratio of 4:1 in clinical samples (15), although the gender ratio has been closer in community observational research (16). Often, the first symptoms experienced are simple motor tics of the eye and face, such as eye blinking. Symptoms might progress in a rostro-caudal direction (i.e., outward from the eyes to other body parts) and to more complex motor tics and vocal tics (5).

In Functional Neurological Symptom Disorder, also referred to as Functional Neurological Disorder (FND), individuals demonstrate neurological symptoms that are not explained by a recognizable illness and might be associated with psychological or physical stress or trauma (17). There are many potential manifestations, the most common of which are altered bodily sensations (e.g., numbness/tingling), loss of balance, tremors, speech difficulties, and muscle jerks (myoclonus) (18, 19). More rarely, symptoms can include tics (20), and the term “functional tic-like behaviors (FTLBs)” has been used to describe these symptoms. In contrast to symptoms of TDs, FTLBs typically have later onset (i.e., teen onset vs. childhood onset), female preponderance, and present as sudden-onset elaborate tic symptoms (5, 6, 21, 22). Additionally, persons with TDs typically have a premonitory sensation—a feeling or sensation that precedes the tics and that is usually relieved by experiencing a tic. Reports suggest people with FTLBs do not experience this sensation (5, 6, 22, 23), although this characteristic has not been systematically studied. Like tics associated with TS, FTLBs are not purposeful or voluntary. Although echo phenomena—repeating movement or words of others (24)—occur frequently among TS patients, they might be more common or pronounced among FTLBs patients (6). Furthermore, FTLBs and tics associated with TS are typically similar in appearance, suggestibility and distractibility, and are exacerbated during periods of stress (5, 23).

The COVID-19 pandemic has led to increased use of the internet, including social media. In a survey of adults conducted in April 2021, 72% of parents with school-aged children (Kindergarten-12^th^ grade) at home said that their children engaged in more screen time than they did before the pandemic (25). Social media use has been associated with poorer mental health among adolescents (26), although it also provides an opportunity to be socially connected while physically distanced. It has been suggested that social media–TikTok in particular–might play a role in increasing prevalence of FTLBs through increased visibility of and exposure to tics (3–5, 8, 27–30).

In October 2021, school staff from a rural area in Minnesota expressed concern that the emergence of tic-like behaviors among students at their school was related to COVID-19 and requested assistance from the Minnesota Department of Health. The objective of this report is to describe the investigation, key findings, and public health implications.

## Methods

On October 15, 2021, the Minnesota Department of Health began investigating a cluster of students who were experiencing tic-like behaviors; the students attended a rural Minnesota high school with approximately 60 students per grade. Initially, school nursing staff reported that six students had FTLB symptoms. Three more students with FTLB symptoms were later reported. Using a standardized questionnaire, we interviewed each affected student and a parent to collect information about the students' symptoms, medical history, social activities, and social media use, and validated depression (Patient Health Questionnaire-9) and anxiety (Generalized Anxiety Disorders-7) screens. For students who visited a healthcare facility for their symptoms, medical records were requested. This activity was reviewed by the Centers for Disease Control and Prevention and was conducted consistent with applicable federal law and Centers for Disease Control and Prevention policy.^1^ Students and their parents provided verbal consent to be interviewed.

A case was defined as rapid onset of FTLBs lasting >24 h during September–November 2021. Rapid onset was defined as abrupt onset of tic-like behaviors with escalation to peak severity within hours to days. Students were referred to mental health professionals for evaluation. All parents and school staff were offered an FTLBs educational webinar.

## Results

The high school reported nine students with tic-like behaviors. Of these, eight had an illness that met the FTLB case definition; one student's symptoms lasted < 24 h after initial report and was not included. Symptom onsets ranged from September 26 to October 30, 2021. All eight students were female (Table 1). Median age at onset was 16 years (range: 15–17 years). Median symptom duration at time of interview was 7 days (range 3–24 days); only one student's symptoms had resolved. Median time between the first and second tic-like symptom was 1.5 days (range: 0–16 days). All eight students reported simple motor tics including head jerks (eight students) and shoulder shrugs (five students). Three students reported eye twitching or blinking, and three reported abdominal tensing. Two students reported complex vocal tics (i.e., syllables or phrases) including one who reported also having simple verbal tics (i.e., nose whistling). One student reported their first tic-like symptom was eye twitching, while the other seven reported neck or head movements as their first symptom. None of the students reported a premonitory sensation. Three students volunteered that their symptoms worsened around others experiencing symptoms.

**Table 1:** Demographic characteristics, symptoms, and medical history of persons experiencing functional tic-like behaviors, Minnesota, September–November 2021.

**Characteristic**	
**Sex—no. (%)**	
Female	8 (100)
**Age—yr**	
Median	16
Range	15–17
**Duration of symptoms at time of interview, days**	
Median	7
Range	3–24
**Duration between new symptoms, days**	
Median	1.5
Range	0–16
**Symptoms—no. (%)**	
Premonitory urge	0 (0)
Simple motor tics	8 (100)
Eye twitching/blinking	3 (38)
Head jerks	8 (100)
Shoulder shrugs	5 (63)
Abdominal tensing	3 (38)
Simple verbal tics	1 (13)
Nose whistling	1 (13)
Complex verbal tics	2 (25)
Syllables, words, phrases	2 (25)
Symptoms worsen around others	3 (38)
Symptoms worsen with stress/anxiety	3 (38)
**Medical History—no. (%)**	
History of COVID-19	4 (50)
Sore throat in 3 months prior to onset	6 (75)
**Psychiatric and neurodevelopmental disorders—no. (%)**	
History of ADHD diagnosis	2 (25)
History of OCD diagnosis	1 (13)
History of anxiety diagnosis	5 (63)
History of depression diagnosis	4 (50)
Current symptoms of anxiety based on GAD-7^*^	7 (88)
Current symptoms of depression based on PHQ-9^*^	5 (71)^†^

^*^More than minimal.

^†^One student refused.

ADHD, attention-deficit/hyperactivity disorder; GAD-7, Generalized Anxiety Disorders-7; OCD, obsessive-compulsive disorder; PHQ-9, Patient Health Questionnaire-9.

None of the students had a previous tic disorder diagnosis. Six (75%) students reported a sore throat < 3 months before FTLB symptom onset. Four students previously had confirmed COVID-19: the interval between prior COVID-19 and tic symptom onset varied: more than a year (one student) ~5 months (one student), and ~2 weeks (one student). One student was diagnosed with COVID-19 when seeking treatment for tic-like behavior. Two other students reported COVID-19-like symptoms in the 2 months before tic symptom onset but did not seek COVID-19 testing. Two students (25%) had a previous diagnosis of attention deficit/hyperactivity disorder (ADHD), one also had a previous diagnosis of obsessive-compulsive disorder (OCD). Five students (63%) had previously been diagnosed with anxiety and four (50%) with depression, including three with a previous diagnosis of both. Using the validated screening tests, five of seven students scored as having more than minimal depression and seven of eight students scored as having more than minimal anxiety. Among students without a previous anxiety or depression diagnosis, respectively, two of three scored as having moderate or severe anxiety, and three of four as having moderate or moderately severe depression. All students had a history of depression or anxiety or scored as having depression or anxiety.

All eight students reported social media use (Table 2). Six students (75%) reported spending >3 h on social media per day. Three students (38%) reported watching videos of tics or TS before symptom onset and two (25%) after symptom onset. However, only one student reported watching the videos more than one or two times. One student reported watching videos before symptom onset on Snapchat. Another student reported watching videos before symptom onset on YouTube and TikTok and another student who had seen videos before could not recall the platform. All eight students could name other students who were also experiencing symptoms. Every student also either participated in extracurricular activities with, or was friends or acquaintances of, one or more other affected students.

**Table 2:** Self-reported social media use and behaviors of persons experiencing functional tic-like behaviors, Minnesota, September–November 2021.

	**No. (%)**
Use social media	8 (100)
**Hours per day**	
< 1 h	0 (0)
1–3 h	2 (25)
3–5 h	4 (50)
5+ h	2 (25)
**Platforms used**	
Facebook	6 (75)
Twitter	0 (0)
Instagram	8 (100)
TikTok	8 (100)
Snapchat	8 (100)
VSCO	3 (38)
Saw videos, posts, stories about tics/Tourette Syndrome *before* symptoms started	3 (38)
Saw videos, posts, stories about tics/Tourette Syndrome *after* symptoms started	2 (25)

## Discussion

The profile of affected students in our investigation showed some consistencies with other reports of FTLBs (1, 3, 5, 8, 23, 28), and differed from the usual presentation of tic disorders in female preponderance, adolescent onset, elaborate tic symptoms on initial presentation, and no premonitory sensation. Notably, we observed all students to have simple tic-like behaviors, which differs somewhat from prior reports that emphasize the presence of highly complex tics. Three presented with eye blinking/twitching, which is typically one of the first organic tics to emerge in TDs, making it hard to entirely rule out a TD etiology in at least some of the students. When initially reported to the Minnesota Department of Health, there was concern that the presentation of tics among students might have been directly related to recent COVID-19. However, only half of the students with onset of FTLBs had prior confirmed COVID-19, and the timing of COVID-19 relative to onset of FTLBs varied markedly. Only two students had current or history of COVID-19. Our investigation does not support that FTLBs are direct sequelae of COVID-19.

The small size of the school in our investigation provided multiple opportunities for affected students to interact. All affected students could name other students experiencing symptoms and were either friends or acquaintances of other affected students or participated in extracurricular activities with one or more affected students. Conversely, only three students had seen videos of tic behaviors on social media prior to onset of symptoms, and only one reported watching them more than one or two times and months before symptom onset. Given the presence of known school/social interactions, and the relative lack of exposure to tic-like behaviors on social media, echo phenomena likely played a greater role in the emergence of FTLBs than social media influence.

The high prevalence of underlying anxiety and depression among the affected students in the cluster supports the hypothesis that tic-like symptoms might be related to underlying mental health disorders or current symptoms of anxiety and depression. In 2019, half of female students reported feelings of sadness or hopelessness (31). The COVID-19 pandemic has exacerbated mental health concerns in children (32, 33), so the observed increases in persons with FTLBs might be multifactorial and an indirect impact of the pandemic related to social isolation, changes in usual structure and routine, increased social media exposure, and other pandemic-related stress among adolescents (2, 34). On December 6, 2021, the U.S. Surgeon General issued a public health advisory to describe and provide recommendations for addressing mental health challenges faced by children and adolescents (32).

It is also notable that some students had a history of ADHD and OCD, which are neurodevelopmental disorders that co-occur with tic disorders at a high rate (15). Tic disorders, ADHD, and OCD have phenotypic similarities (e.g., repetitive behaviors, motor disinhibition) that are thought to result from common genetic vulnerability (35) and converging dysfunction of cortico-stratial circuits (36). This raises the possibility that some FTLBs emerge in the context of an underlying biological susceptibility that is “tic disorder-like” in nature. For example, individuals with more active or strongly connected sensorimotor neurocircuitry might have a greater propensity toward echo phenomena (37) and habit formation of motor sequences (38). There is a pressing need for research to identify the neurobiological mechanisms involved in FTLBs, including how these mechanisms might converge or diverge with those driving tics and socially-mediated tic exacerbations.

This investigation had a few notable limitations. First, this investigation included only passive case identification since tic-like behaviors are susceptible to echo phenomena. As a result, cases might have been missed. However, since the school is relatively small, it is unlikely other cases would have gone unnoticed. Second, although we requested medical records for the four students who sought medical care for their symptoms, we were only able to obtain records for two students which limited the ability to compare treatments.

Clinicians have recognized that there might be sex-specific differences in the clinical presentation and subsequent treatment of tics (39). There is some evidence that tics associated with tic disorders in females might be more complex, begin later in life, and co-occur with mood and anxiety disorders (39). FTLBs appear to occur more frequently among females (1, 4, 6, 23) however, the recognition that tic presentation might be different among females highlights an area where more research is needed to understand how tic disorders might vary among females. Our findings highlight the need for a better understanding of FTLBs and mental health in adolescents, especially in the context of the COVID-19 pandemic.

## Data availability statement

The raw aggregate data supporting the conclusions of this article will be made available by the authors, without undue reservation.

## Ethics statement

Ethical review and approval was not required for the study on human participants in accordance with the local legislation and institutional requirements. Written informed consent from the students and their legal guardian was not required to participate in this study in accordance with the national legislation and the institutional requirements.

## Author contributions

MF led the investigation, designed the data collection tool, collected data, drafted the initial manuscript, and critically reviewed and revised the manuscript. SH, CC, RD, KS, RB, RL, SK, and SG guided development of the data collection instrument and critically reviewed and revised the manuscript. All authors contributed to the article and approved the submitted version.

## Footnotes

^1^45 C.F.R. part 46.102(l)(2), 21 C.F.R. part 56; 42 U.S.C. Sect. 241(d); 5 U.S.C. Sect. 552a; 44 U.S.C. Sect. 3501 et seq.
